# Linking Oxidative Stress to Placental Dysfunction: The Key Role of Mitochondria in Trophoblast Function

**DOI:** 10.3390/medsci14010053

**Published:** 2026-01-21

**Authors:** Ioanna Vasilaki, Anastasios Potiris, Efthalia Moustakli, Despoina Mavrogianni, Nikoletta Daponte, Theodoros Karampitsakos, Alexios Kozonis, Konstantinos Louis, Christina Messini, Themos Grigoriadis, Ekaterini Domali, Sofoklis Stavros

**Affiliations:** 1 Medical School, National and Kapodistrian University of Athens, 11527 Athens, Greece; jeannettevas@windowslive.com; 2 Third Department of Obstetrics and Gynecology, University General Hospital “ATTIKON”, Medical School, National and Kapodistrian University of Athens, 12462 Athens, Greece; theokarampitsakos@hotmail.com (T.K.); alkozonis@gmail.com (A.K.); kostaslouisss@gmail.com (K.L.); sfstavrou@med.uoa.gr (S.S.); 3 Department of Nursing, School of Health Sciences, University of Ioannina, 45500 Ioannina, Greece; ef.moustakli@uoi.gr; 4 First Department of Obstetrics and Gynecology, Alexandra Hospital, Medical School, National and Kapodistrian University of Athens, 11528 Athens, Greece; dmavrogianni@med.uoa.gr (D.M.); tgregos@med.uoa.gr (T.G.); kdomali@yahoo.fr (E.D.); 5 Department of Obstetrics and Gynecology, Faculty of Medicine, School of Health Sciences, University of Thessaly, 41500 Larissa, Greece; nikolettadaponte@gmail.com (N.D.); messini@uth.gr (C.M.)

**Keywords:** oxidative stress (OS), mitochondrial dysfunction, reactive oxygen species (ROS), redox homeostasis, trophoblast dysfunction, pregnancy complications

## Abstract

Oxidative stress (OS) is a critical regulator of placental development; however, its specific effects on trophoblast biology remain incompletely elucidated. This narrative review synthesizes evidence derived from studies using human placental tissues and trophoblast cell models to delineate how excessive reactive oxygen species (ROS) disrupt molecular and cellular pathways essential for normal placentation. The literature search was restricted to human-based and in vitro investigations. Across these studies, OS was consistently shown to impair mitochondrial function in trophoblasts, resulting in increased mitochondrial ROS generation, loss of mitochondrial membrane potential, and activation of apoptotic signaling cascades. These mitochondrial disturbances were associated with reduced trophoblast proliferation, migration, and invasion, as well as dysregulation of angiogenic balance. Furthermore, several studies reported alterations in mitophagy, involvement of redox-sensitive pathways such as CYP1A1 and KLF9, and the extracellular release of mitochondrial DNA, which was linked to reduced cell viability and increased necrotic cell death. Collectively, the available evidence indicates that OS interferes with key trophoblast-dependent developmental processes, providing mechanistic insight into the pathogenesis of placental dysfunction observed in pregnancy complications such as preeclampsia (PE) and intrauterine growth restriction (IUGR). Elucidation of these pathways may inform the development of targeted therapeutic strategies aimed at preserving placental function and improving adverse pregnancy outcomes.

## 1. Introduction

The placenta functions as a special organ which creates a connection between the mother and her developing fetus to enable pregnancy through its ability to exchange nutrients and gases and produce hormones and maintain immune tolerance. It is the first and largest fetal organ that develops and undergoes continuous structural and functional remodeling throughout gestation while supporting fetal growth. Proper placental development is essential for a successful pregnancy, whereas abnormal placentation leads to serious consequences for both the fetus and the mother. Research findings show that early placental development problems lead to permanent changes because they determine the pregnancy outcome. These observations underscore that placental dysfunction starts during early pregnancy and it becomes the main factor which causes various pregnancy-related complications [1].

OS arises when the production of ROS exceeds the capacity of endogenous antioxidant systems to neutralize them and repair the resulting cellular damage [2]. The process of cellular metabolism produces ROS as metabolic byproducts which mainly occur during mitochondrial oxidative phosphorylation. The body maintains a balance between reactive species under normal conditions which enables redox signaling to perform its role in supporting cellular operations. The body maintains its natural state through mitochondrial function and proper ROS-generating enzyme regulation and sufficient antioxidant defenses and environmental stress protection. The body develops OS when its natural equilibrium between antioxidants and free radicals becomes unbalanced because of mitochondrial dysfunction or enzyme dysregulation or decreased antioxidant defenses or environmental stress exposure which leads to lipid and protein and nucleic acid damage [3].

The placental tissue faces an increased risk of OS because it maintains elevated metabolic activity to support fetal development during pregnancy. Controlled ROS production is required to perform its physiological functions which include trophoblast invasion, placental remodeling, vascular development and early organogenesis. The first trimester of development needs particular ROS-generating systems which function at elevated levels in early placental tissues to enable proper placental development. Excessive or poorly regulated OS leads to abnormal placental development, immune system imbalance, and placental tissue damage. The condition leads to multiple pregnancy complications which include recurrent pregnancy loss, PE, IUGR, and preterm birth [4].

Mitochondria occupy a central position at the intersection of placental metabolism and OS. The mitochondria function as the main location for oxidative phosphorylation while producing ROS but they control redox signaling pathways which occur under physiological conditions. In the placenta, mitochondrial activity must adapt to changing oxygen availability and metabolic demands throughout gestation in order to allow trophoblasts to sustain energy production while limiting excessive ROS generation [5]. However, when mitochondrial activity becomes dysregulated, ROS accumulate to pathological levels, shifting redox balance toward OS [6].

The excessive production of OS stress leads to trophoblast dysfunction which results in poor spiral artery development and reduced placental blood flow that causes PE and IUGR. In PE, intermittent hypoxia–reoxygenation episodes increase oxidative damage while releasing antiangiogenic factors that include soluble Flt-1 and soluble endoglin, which drive systemic endothelial dysfunction. The placental hypoxia and OS in IUGR establish permanent conditions which prevent vital nutrients and oxygen from reaching the fetus thus stopping its development. The disruption of cellular OS between autophagy and apoptosis survival pathways leads to placental dysfunction and insufficient placental function [7].

Extensive evidence of human studies has proven that human exposure to OS causes placental damage. This topic is continuously studied through separate investigations which examine particular stress factors and biological targets. Many studies have examined these stressors separately but it needs to be determined how different types of OS affect mitochondrial pathways which control trophoblast function. The research lacks a comprehensive analysis of how mitochondrial redox imbalance functions as a unifying mechanism which connects various trophoblast models under different stress conditions. The absence of an integrated framework prevents uniting different studies because it blocks their ability to detect common mitochondrial elements which cause placental problems.

In this review, we integrate evidence in order to examine how different forms of OS shape trophoblast function. We focus on mitochondrial dysfunction as a central organizing principle linking redox imbalance to impaired trophoblast invasion, survival, angiogenic signaling, and inflammatory activation. By synthesizing mechanistic insights across experimental models and pathological contexts, this review aims to provide a coherent, mitochondria-centered framework for understanding OS-driven placental dysfunction in pregnancy complications.

## 2. Materials and Methods

This study was conducted as a narrative review using a structured, yet non-systematic, methodological approach. Literature searches were performed using PubMed, Scopus, and Google Scholar. The search strategy incorporated the keywords “oxidative stress,” “reactive oxygen species,” “trophoblasts,” “trophoblastic cells,” “hypoxia,” and “placenta,” combined using Boolean operators (AND/OR) in various configurations. To ensure comprehensive coverage, a snowball searching approach was additionally employed, whereby reference lists of eligible articles were screened to identify further relevant studies.

Studies were excluded if they involved animal models, clinical or experimental drug administration, therapeutic intervention protocols, or viral exposure or infection models. Additional exclusion criteria included conference abstracts, case reports, purely epidemiological studies, non-English publications, and studies lacking mechanistic investigation in human trophoblast-related systems. Articles focusing exclusively on clinical biomarkers without accompanying functional or molecular experimentation were also excluded.

Given the narrative scope of this review, no PRISMA flow diagram, quantitative meta-analysis, or formal statistical synthesis was performed. Furthermore, a structured risk-of-bias or quality assessment was not undertaken, as the primary objective of this review was to provide an integrated mechanistic overview of OS-related pathways in trophoblast biology rather than to derive quantitative effect estimates.

Selection bias and inadequate coverage of all pertinent studies are inherent risks associated with the narrative technique used in this evaluation. However, given known species-specific variations in placentation and mitochondrial regulation, limiting inclusion to human placental tissues and in vitro trophoblast models was a purposeful tactic to improve mechanistic relevance and translational consistency. A targeted synthesis of cellular and molecular pathways directly controlling trophoblast OS responses is made possible by excluding animal models, therapeutic treatments, and epidemiological studies; however, this restricts the capacity to deduce causality or therapeutic efficacy. To validate and expand on these findings, future integrative investigations incorporating both in vivo and clinical data will be necessary. As a result, this study prioritizes mechanistic insight above quantitative generality.

## 3. Results

### 3.1. Mitochondrial Dysfunction and Redox Imbalance in PE

PE is frequently associated with placental mitochondrial dysfunction and OS, which may contribute to trophoblast impairment. Several studies report that PE placentas exhibit mitochondrial abnormalities, including decreased mitochondrial content, reduced mtDNA copy number, lower citrate synthase activity, and reduced levels of mitochondrial-encoded electron transport chain (ETC) components such as COXII. These alterations are accompanied by increased expression of glycolytic enzymes (HKII, PFK) and glucose transporter 1 (GLUT-1), consistent with metabolic compensation. The mitochondrial biogenesis process depends on PGC-1α/β and other key regulators which show decreased expression but NRF1 and ERRα protein levels are increased, possibly reflecting compensatory signaling.

Vangrieken et al. [8] reported altered mitochondrial quality control, with increased BCL2 interacting protein 3 (BNIP3) and BCL2 interacting protein 3-like (BNIP3L) but unchanged PTEN-induced kinase 1 (PINK1) and Parkin (PARK2) (PINK1/Parkin), alongside increased mitochondrial fission marked by elevated dynamin 1-like protein (DNM1L). These changes occur because of increased ROS, an imbalanced antioxidant response, characterized by increased manganese superoxide dismutase 2 (MnSOD2), reduced catalase activity, and an increased BCL2-associated X protein (BAX)/B-cell lymphoma 2 (BCL-2) ratio. Together, these findings support enhanced apoptosis and inflammatory activation in PE placentas.

Another study established in vitro PE-like models using primary cytotrophoblasts (CTBs) isolated from healthy term placentas and exposed to either hypoxia or hypoxia/reoxygenation (H/R). Clinically, PE pregnancies were characterized by higher maternal blood pressure and proteinuria and smaller neonatal birth measurements. PE placental tissue showed increased mRNA expression of angiogenic and antiangiogenic factors (FLT-1, ENG, VEGF-A), along with reduced AGTR4 and NRF2 expression. Selected ER stress markers (GRP78, GRP94) were decreased, whereas inflammasome-related markers (NLRP3, IL-1β, CASP1) and hCG were increased.

In vitro, both hypoxia and H/R reduced PIGF released, increased malondialdehyde (MDA), induced ER stress markers, and enhanced inflammasome activation. The sFLT-1/PlGF ratio in both models exceeded clinical PE thresholds, supporting their relevance to PE-associated placental stress. Importantly, hypoxia and H/R elicited distinct responses: hypoxia primarily affected angiogenic and differentiation marker expression, whereas H/R more closely reproduced OS-associated features [9].

Early placental dysfunction in PE is also linked to altered mitochondrial gene programs. Yu et al. [10] used patient-derived induced pluripotent stem cells (iPSCs) from human amniotic epithelial cells (hAECs) to generate trophoblast stem-like cells (TSLCs). These TSLCs recapitulated features consistent with early-onset PE, including associations with low birth weight and hypertensive phenotypes. Transcriptomic profiling showed decreased expression of nucleoside diphosphate kinase 4 (NME4) and other mitochondrial matrix-related genes, consistent with mitochondrial dysfunction and increased OS. The authors also reported increased p53 and apoptosis-associated signals, alongside increased thioredoxin (TRX), suggesting activation of compensatory antioxidant defenses.

In maternal peripheral blood mononuclear cells (PBMNCs), NME4 expression peaked during late preterm stages, indicating dynamic regulation across gestation. Together, these findings support a link between reduced NME4-associated mitochondrial homeostasis, trophoblast injury, and OS in PE, although the mechanistic contribution of reduced NME4 requires further clarification. Notably, these results come from distinct experimental systems (primary trophoblasts, patient tissues, and TSLCs), and interpretation should consider differences in trophoblast subtype, gestational stage, and stress exposure. Collectively, the available evidence indicates that OS-related mitochondrial dysfunction engages multiple regulatory nodes that shape trophoblast viability and broader cellular stress responses [11,12]. Overall, these studies indicate that PE is characterized by qualitative mitochondrial dysfunction in trophoblasts, marked by impaired bioenergetics, excessive ROS generation, altered mitochondrial quality control, and activation of apoptotic and inflammatory pathways.

### 3.2. Hypoxia-Driven Metabolic and Cholesterol Dysregulation in Trophoblasts

Beyond structural mitochondrial defects, hypoxia profoundly alters trophoblast metabolism, particularly lipid and cholesterol homeostasis. In primary trophoblasts, hypoxia and H/R promote accumulation of non-esterified cholesterol. This is associated with increased expression of cholesterol biosynthesis enzymes, including 3-hydroxy-3-methylglutaryl-CoA reductase (HMGCR) and 3-hydroxy-3-methylglutaryl-CoA synthase 1 (HMGCS1), together with reduced acetyl-CoA acetyltransferase 1 (ACAT1) activity. Low-density lipoprotein (LDL) uptake via the low-density lipoprotein receptor (LDLR) appears largely preserved.

Hypoxia also increased expression of sterol regulatory element-binding protein 2 (SREBP-2) and sterol regulatory element-binding protein 1a (SREBP-1a), linking sterol regulatory transcription to oxygen-dependent signaling. In parallel, hypoxia-inducible factor 2 alpha (HIF-2α) protein levels increase, whereas hypoxia-inducible factor 1 alpha (HIF-1α) decreases. Cholesterol release into the maternal and fetal circulation becomes more efficient through ATP-binding cassette subfamily G member 1 (ABCG1) and scavenger receptor class B type I (SR-BI). However, SR-BI and ABCA1 protein levels decrease despite increased mRNA expression.

ROS levels rise without immediate cytotoxicity, consistent with an OS phenotype.

These findings are mirrored in PE placentas, which show increased non-esterified cholesterol, increased cholesterol biosynthesis enzymes, and increased ABCG1 and SR-BI expression. Together, the data implicate hypoxia-driven cholesterol dysregulation as a mechanism linking placental stress to PE pathophysiology [13]. These metabolic changes likely interact with mitochondrial bioenergetic impairment and ROS amplification, consistent with broader trophoblast stress reprogramming.

Analysis of placentas from PE pregnancies showed reduced sirtuin 3 (SIRT3) expression in trophoblasts, which is associated with mitochondrial damage and increased ROS. Reduced SIRT3 promotes MnSOD hyperacetylation and inactivation and decreases ATP production, indicating impaired mitochondrial redox buffering. In vitro, SIRT3 deficiency increases OS and inflammatory factor production, whereas SIRT3 overexpression restores mitochondrial function and decreases ROS. Collectively, these findings support a protective role for the SIRT3–MnSOD axis in maintaining trophoblast mitochondrial stability under OS in PE [14]. Together, these findings suggest that hypoxia-driven metabolic and cholesterol dysregulation contributes to mitochondrial stress and oxidative imbalance in trophoblasts, thereby linking altered placental metabolism to the pathophysiology of PE.

### 3.3. Redox-Sensitive Genes Regulating Trophoblast Survival and Dysfunction

Several redox-sensitive genes have been implicated as regulators of trophoblast survival, senescence, and invasion under OS conditions. Phosphoglucomutase 5 (PGM5) transcripts are detected throughout gestation but are markedly reduced in PE and IUGR placentas, and placental PGM5 expression correlates with fetal birth weight. In primary cytotrophoblasts, PGM5 silencing increased the expression of genes linked to apoptosis, growth signaling, and oxidative stress, including BAX, EGFR, IGF2, and NOX4, under both normoxic and hypoxic conditions. NOX4 upregulation after PGM5 knockdown supports increased pro-oxidant signaling when PGM5 is reduced. Collectively, these data suggest that reduced PGM5 contributes to trophoblast dysfunction by amplifying OS-responsive gene programs [15].

The nuclear receptor subfamily 4 group A member 2 (NR4A2) transcripts are elevated in pregnancies complicated by PE with IUGR. However, placental NR4A2 expression is not altered in preterm pathological placentas and appears increased only at term. In primary cytotrophoblasts, hypoxia markedly reduces NR4A2 mRNA, whereas protein levels remain unchanged. NR4A2 silencing under hypoxia does not affect trophoblast survival or the production of angiogenic factors (sFlt-1, PlGF). By contrast, hypoxia is associated with upregulation of OS and inflammatory genes, including NOX4, HMOX-1, GCLC, NLRP3, and SPINT1. Overall, NR4A2 appears to modulate oxidative and inflammatory stress responses in hypoxic trophoblasts, with limited effects on angiogenic imbalance [16].

Advanced oxidation protein products (AOPPs) induce a senescence-like phenotype in HTR-8/SVneo trophoblast cells through OS. AOPP exposure increases senescence markers, including SA β-gal activity, SAHF formation, mitochondrial membrane depolarization, and G0/G1 cell-cycle arrest. N-acetylcysteine (NAC) reverses these effects, supporting an OS-dependent mechanism. AOPPs also impair autophagic flux with reduced LC3-positive autophagosomes/autolysosomes, decreased BECN1, and accumulation of p62. Rapamycin-induced autophagy partially attenuates AOPP-induced senescence. Mechanistically, AOPPs activate the p53–p21 axis and mTOR/p70S6K signaling, linking OS to cell-cycle arrest and autophagy inhibition. Restoration of p53 function rescues mitochondrial function, autophagy, and cell-cycle progression, implicating p53–mTOR signaling as a central pathway in AOPP-induced trophoblast senescence [17].

Heat shock protein family B (small) member 8 (HSPB8) expression is significantly downregulated in placental tissues from patients with preterm PE and in H/R-treated HTR-8/SVneo cells. Restoring HSPB8 improves trophoblast proliferation, migration, and invasion, while reducing OS and apoptosis. H/R increases intracellular ROS and MDA, induces mitochondrial membrane depolarization, and promotes apoptotic signaling through decreased Bcl-2 and increased Bax and cleaved caspase-3 levels. These effects are reversed by HSPB8 overexpression. The protective effects of HSPB8 are reduced when c-Myc is silenced, suggesting that HSPB8 acts, at least in part, through c-Myc-dependent signaling [18].

Neprilysin is upregulated in PE placentas and promotes OS-mediated trophoblast dysfunction. Neprilysin levels correlate positively with blood pressure in PE. In extravillous trophoblasts (EVTs), H_2_O_2_ increases neprilysin expression and ROS accumulation, leading to mitochondrial damage, apoptosis, inflammation, and reduced migration and invasion; neprilysin silencing reverses these effects. The RNA-binding protein insulin-like growth factor 2 mRNA-binding protein 1 (IGF2BP1), which upregulates in PE and under OS conditions, forms a stable complex with neprilysin mRNA to produce elevated protein levels. IGF2BP1 knockdown reduces OS, restores mitochondrial function, and improves trophoblast performance, and these benefits are lost when neprilysin is reexpressed [19].

Rho family GTPase 3 (RND3) is downregulated in PE placentas and trophoblasts and plays a critical protective role against mitochondrial OS. Reduced RND3 is associated with increased ROS, enhanced apoptosis, and greater sensitivity to hypoxic stress. PE trophoblasts show severe mitochondrial damage, including loss of cristae structures and collapse of membrane potential. RND3 overexpression reduces ROS, restores membrane potential, and decreases cell death, whereas RND3 knockdown promotes mitochondrial uncoupling and proton leak. Mechanistically, RND3 binds peroxisome proliferator-activated receptor gamma (PPARγ) and stabilizes its nuclear localization, promoting transcriptional activation of uncoupling protein 2 (UCP2). Restoration of PPARγ signaling rescues mitochondrial function and reduces OS under RND3 deficiency. Delivering RND3 through adenovirus to PE primary trophoblasts normalizes ROS levels while mitochondrial function improves and the PPARγ/UCP2 signaling pathway regains its function which confirms the clinical relevance of the ND3–PPARγ/UCP2 axis [20].

The expression of mitochondrial ferritin (FtMt) is upregulated in PE patient placentas while HIF-1α and vascular endothelial growth factor (VEGF) protein levels also increase. The HTR-8/SVneo cells which experienced hypoxia presented proliferation, enhancing invasion and angiogenic capacity along with increased HIF-1α and VEGF protein expression. Reducing FtMt decreases HIF-1α/VEGF signaling under hypoxia and suppresses invasion and tube formation. These findings suggest that FtMt modulates hypoxia-associated trophoblast behaviors through the HIF-1α/VEGF pathway [21].

MiR-141-3p is increased in PE placental tissues and is induced by hypoxia in JEG-3 cells. PE placentas show increased LC3-II and Beclin1 with reduced p62, consistent with enhanced autophagy. In vitro, hypoxia increases autophagosome and autolysosome formation, indicating increased autophagic flux. MiR-141-3p overexpression enhances hypoxia-induced autophagy, whereas miR-141-3p inhibition suppresses LC3-II and Beclin1 and increases p62. Overall, miR-141-3p acts as a hypoxia-responsive regulator of trophoblast autophagy relevant to PE [22].

PE placentas show reduced trophoblast differentiation markers (Syncytin-1/2, SLC1A5, MFSD2A, dysferlin), reduced invasive proteases (MMP-2, MMP-9, uPA), and increased protease inhibitors (TIMP-1, TIMP-2, PAI-1). Ultrastructural analyses reveal extensive mitochondrial damage and ER swelling, consistent with increased OS, lipid peroxidation, and altered antioxidant enzyme activity. In trophoblast cell models, non-lethal H_2_O_2_ increases ROS and disrupts differentiation marker expression while altering migratory behavior. OS activates the IRE1α–XBP1s arm of the unfolded protein response in both HTR-8/SVneo and BeWo cells. NAC pretreatment attenuates these effects, supporting a direct contribution of OS to impaired trophoblast differentiation/invasion programs and ER homeostasis in PE [23]. Overall, these data highlight that multiple redox-sensitive genes modulate trophoblast survival, senescence, invasion, and angiogenic capacity by shaping mitochondrial function and OS responses under pathological conditions.

### 3.4. OS, Inflammation, and Autophagy–Inflammasome Crosstalk

The HTR-8/SVneo trophoblast cells developed severe OS when exposed to H/R which led to increased ROS production and reduced activities of CAT, SOD, and GSH-Px enzymes. The Keap1–Nrf2 axis maintains trophoblast redox balance, as evidenced by the finding that Nrf2 silencing increased ROS levels and reduced antioxidant enzyme activity. The placental tissues obtained from preeclamptic pregnancies showed decreased Keap1 expression but Nrf2 and HO-1 levels increased substantially at both mRNA and protein levels despite having low antioxidant enzyme activity. The Keap1-Nrf2-HO-1 pathway activates its protective mechanisms through enhanced activity because PE induces OS which damages the placenta [21].

The protein PRDX1 was highly expressed in normal placental trophoblast cells but its expression became significantly downregulated in preeclamptic placental tissue and H_2_O_2_-exposed HTR-8/SVneo cells. Zhou M. et al. [22] showed that OS caused PRDX1 protein levels to decrease while it led to increased ROS production and elevated levels of autophagy markers Beclin1 and LC3-II. PRDX1 knockdown exacerbated H_2_O_2_-induced defects in trophoblast migration, invasion, and tube formation, suppressed proliferation, and promoted apoptosis via upregulation of Bax and cleaved caspase-3. PRDX1 regulated autophagy through the PTEN/AKT pathway while its loss led to increased ROS levels and apoptosis, both of which were partially reversed by the antioxidant NAC.

The *MGST1* gene is significantly downregulated in preeclamptic placental tissue but it functions as a protective mechanism against hypoxic damage in trophoblast cells. In vitro, it was proved that OS which occurred under hypoxic conditions decreased *MGST1* gene expression but *MGST1* overexpression restored antioxidant capacity through elevated SOD and GSH production and decreased MDA and MPO levels. *MGST1* functionally promoted trophoblast proliferation, migration, and invasion under hypoxic conditions. The PI3K/AKT/mTOR signaling pathway was activated through *MGST1* which resulted in enhanced phosphorylation of pathway components. Pharmacological inhibition of PI3K abolished the protective effects of *MGST1* on OS and trophoblast function. These findings identify *MGST1* as a vital redox-sensitive factor which controls trophoblast cell behavior through PI3K/AKT/mTOR signaling pathways [23].

Hypoxia and hypoxia–reoxygenation markedly impaired trophoblast function by increasing intracellular ROS, reducing SOD activity, and suppressing migration and invasion in HTR-8/SVneo cells. These alterations were accompanied by extensive epigenetic modifications, including numerous differentially methylated regions. DNMT1 and DNMT3A expression levels were substantially reduced, suggesting that OS drives widespread changes in DNA methylation patterns. The integrated methylome–transcriptome analysis revealed that vital metabolic and signaling pathways including MAPK and axon guidance pathways experience coordinated regulatory changes. The genes PFKFB3 and ANKRD37 demonstrated hypoxia-triggered promoter hypomethylation which led to increased gene expression while showing similar changes to placental tissue from preeclamptic pregnancies. The research establishes a direct connection between OS which causes epigenetic changes that result in gene expression abnormalities in trophoblast cells which contribute to PE development [24].

Loss of STOX1 profoundly alters trophoblast proliferation, fusion, and OS responses. The STOX1 knockout in BeWo cells resulted in enhanced cAMP activation which led to increased expression of fusion markers Syncytin-2 and CYP19A1 and CGB and disrupted E2F-dependent cell-cycle regulation. STOX1 serves as an essential factor which maintains redox homeostasis because knockout cells displayed increased basal superoxide production. The Y153H which appears in disease-associated variants enhanced proliferative capacity but the R364X truncated mutant presented high sensitivity to OS which caused apoptosis through vacuolization upon H_2_O_2_ exposure. The wild-type cells and STOX1-rescued cells maintained their trophoblast fusion process under OS but the cells with pathogenic variants lost all fusion ability. The genetic factor STOX1 emerges as a critical regulator which controls trophoblast cell survival and differentiation under OS conditions [25].

FtMt expression is upregulated in placentas from PE patients alongside increased HIF-1α and VEGF levels. Under hypoxic conditions, HTR-8/SVneo cells showed suppressed proliferation but enhanced invasion and angiogenic capacity, accompanied by increased HIF-1α and VEGF expression. FtMt overexpression downregulated hypoxia-induced HIF-1α/VEGF signaling and reduced invasion and tube formation, whereas FtMt silencing restored these effects, indicating that FtMt acts as a negative regulator of hypoxia-driven trophoblast angiogenic and invasive responses via the HIF-1α/VEGF pathway [18]. In summary, OS promotes a tightly interconnected network involving impaired antioxidant defenses, dysregulated autophagy, and inflammasome activation, which together exacerbate trophoblast dysfunction in PE.

### 3.5. Metabolic, Environmental, and Maternal Stressors Inducing Placental OS

Hyperglycemia in pregnancy (HIP), including gestational (GDM) and pregestational diabetes (PGDM), significantly impacts placental energy metabolism and OS responses through mitochondrial and gene regulatory mechanisms. The trophoblasts from HIP pregnancies showed impaired mitochondrial function which resulted in abnormal ATP generation and elevated ROS production that caused redox imbalance and apoptosis. The AMPKα–p38MAPK signaling pathway links cellular energy sensing to OS responses and GLUT1 transporter expression. The downstream transcription factors Trp53 and HIF-1α and Myc and Mknk2 were implicated in inducing these metabolic and stress-related gene programs, which potentially regulate apoptosis, OS, and metabolism. The study highlights that HIP-induced mitochondrial dysfunction and gene regulatory imbalances result in placental maladaptation, suggesting that targeted modulation of mitochondrial activity or AMPKα-dependent transcriptional networks could provide therapeutic strategies to protect both maternal and fetal outcomes [26].

COLEC12 shows increased expression in insulin-resistant HTR-8/SVneo cells which results in impaired glucose metabolism, OS, and dysregulated insulin signaling. COLEC12 overexpression leads to decreased glucose consumption because it enhances GLUT1 expression and decreases GLUT4 distribution while knockdown reversed these effects. The modulation was associated with improved insulin signaling because COLEC12 knockdown produced higher levels of p-IRβ and p-IRS-1 and p-AKT proteins which simultaneously reduced p-IRS-1 (Ser307), promoting glucose uptake. Moreover, COLEC12 regulated OS by modulating ROS accumulation and SOD activity which highlighted a connection between metabolic dysfunction and mitochondrial stress responses. In addition, it influenced the COX-2/PGE2 inflammatory axis, with knockdown reducing proinflammatory signaling and enhancing glucose metabolism, highlighting a gene–mitochondria–inflammation network in the pathogenesis of gestational diabetes. The research indicates that COLEC12 targeting could help restore mitochondrial function, redox balance, and insulin sensitivity in the placenta which might lead to a potential treatment method for GDM [27].

Placental tissues obtained from GDM pregnancies exhibited significantly increased expression of the OS adaptor protein p66Shc and the mitochondrial fission regulator Drp1. The JEG-3 trophoblast cells showed increased p66Shc and Drp1 protein expression which directly linked to the severity and length of hyperglycemia based on the in vitro study. The cells produced higher amounts of cellular ROS because of the increased p66Shc and Drp1 protein expression. Functional manipulations of p66Shc demonstrated that it has a regulatory role because Drp1 expression and ROS production increased when p66Shc levels were elevated but decreased when p66Shc was knocked down. Huang TT et al. identified a signaling pathway which connects high blood sugar to mitochondrial damage and OS through p66Shc-Drp1-ROS mechanisms in diabetic placental tissue damage [28].

First-trimester placental tissues from spontaneous abortions are characterized by increased oxidative stress markers and enhanced proapoptotic activity. The p53 protein shows increased expression while Bcl-2 antiapoptotic protein levels decrease, suggesting impaired trophoblast survival and differentiation. Antioxidant defenses, including glutathione (GSH) and MTH1, are markedly reduced in spontaneous abortion cases. These changes are associated with chorangiosis and accelerated villous maturation, indicating early ischemic alterations. The placentas exhibit mesenchymal villi with reduced Hofbauer cells and fetal vascular elements and a dual-layered trophoblast epithelium which shows cytotrophoblast prominence during early pregnancy. Statistical analyses confirm that diminished antioxidant capacity together with increased proapoptotic signaling are strongly associated with spontaneous abortion through their impact in early pregnancy failure [29].

The placental tissue of women with hypertensive disorders of pregnancy shows elevated TNFα levels and decreased NRF2 protein amounts which results in impaired antioxidant defense mechanisms. In JEG-3 trophoblast cells, TNFα suppressed NRF2 nuclear localization, increasing mitochondrial superoxide production and oxygen consumption that resulted in mitochondrial OS and bioenergetic overactivation. RvD2 compound activates NRF2 signaling which results in elevated intracellular glutathione levels and NRF2 nuclear translocation during inflammatory conditions. Resolvin D2 (RvD2) prevented TNFα from generating mitochondrial ROS during its operation while it decreased TNFα-induced increases in both resting and peak cellular respiration without affecting electron transport chain protein levels. RvD2 treatment increased mitochondrial mass and membrane potential of TNFα-exposed trophoblast cells but TNFα exposure alone caused substantial migration impairment in trophoblast cells [30].

The first-trimester HTR-8/SVneo trophoblast cells developed hypoxia and OS when exposed to MEHP at different concentrations which produced a dose-dependent response. The cells showed increased ROS levels and HIF-1α activation which led to its nuclear translocation and enhanced VEGF-A expression. The MEHP treatment led to increased expression of miR-210-3p which responds to hypoxia through epigenetic mechanisms. At the mitochondrial level, MEHP leads to major decreases in cellular ATP production, reduced mtDNA content, decreased expression of NDUFB8 and SDHB respiratory chain subunits, and damaged mitochondrial membrane potential. These alterations were accompanied by reduced protein amounts of ETC complexes II–IV which proved that MEHP disrupts mitochondrial bioenergetics and causes mitochondrial dysfunction in trophoblast cells [31].

LAT1 is highly expressed in trophoblast cells and plays a critical role in regulating redox homeostasis during toxic stress. The HTR-8/SVneo cells showed decreased glutathione (GSH) levels after LAT1 gene silencing through siRNA which aggravated methylmercury-induced cytotoxicity and apoptosis while it decreased the amount of intracellular mercury. The depletion of LAT1 protein led to decreased total and reduced GSH levels which were not affected by toxic exposure and produced a sustained decrease in the GSH/GSSG ratio, indicating baseline OS vulnerability. Moreover, amino acid availability strongly modulated this response, as combined cysteine and methionine deficiency severely impaired GSH synthesis and further lowered the GSH/GSSG ratio, whereas amino acid supplementation partially preserved redox balance. The study demonstrates that LAT1-dependent amino acid transport serves as a vital mechanism for trophoblast antioxidant capacity and directly links amino acid metabolism to OS [32].

Placentas from advanced maternal age (AMA) pregnancies exhibit a senescent phenotype, characterized by increased SA-β-gal staining and elevated p53 and p21 expression, along with reduced YAP protein levels and increased oxidative DNA damage, as indicated by 8-OHdG accumulation. The HTR-8/SVneo trophoblast cells developed senescence when H_2_O_2_ caused OS which led to p53/p21 protein persistence, YAP protein reduction, and DNA damage accumulation. Functional studies showed that YAP knockdown alone was sufficient to induce senescence, enhance oxidative DNA damage, and impair trophoblast migration and invasion. On the contrary, YAP overexpression partially protected against H_2_O_2_-induced senescence and DNA oxidation. The YAP knockdown caused changes in the expression of genes involved in DNA damage and telomere stress pathways according to transcriptomic profiling results [33].

BeWo cell culture showed three major effects from hypoxia which included decreased mitochondrial content, loss of mitochondrial membrane potential, and a transition to glycolysis with elevated lactate production. These alterations were accompanied by the suppression of key regulators of mitochondrial biogenesis which included PGC-1β and TFAM and NRF2α and ERRα. The study showed that hypoxia caused changes in both mitophagy processes and mitochondrial structure through increased BNIP3/BNIP3L expression and decreased MFN1 and MFN2 fusion-related gene expression. Together, these findings indicate that hypoxia causes mitochondrial dysfunction which leads to metabolic reprogramming in trophoblast cells through ROS-dependent impairment of mitochondrial homeostasis [34].

In an in vitro inflammatory model of trophoblast dysfunction, KLF9 functions as the key regulator which controls OS-induced pyroptosis. LPS and ATP stimulation of HTR-8/SVneo cells induced a time- and dose-dependent upregulation of KLF9, accompanied by reduced cell viability and impaired migration and invasion. The KLF9 gene silencing restored trophoblast viability and motility while reducing OS, as evidenced by suppressed ROS and MDA levels and by increased SOD and catalase activity. The antioxidant enzyme PRDX6 was negatively regulated by KLF9 and PRDX6 knockdown eliminated all the protective effects from KLF9 gene silencing. Also, KLF9 activated the NLRP3 inflammasome to induce pyroptotic signaling which produced caspase-1 activation and GSDMD and IL-1β and IL-18 release but these effects were reversed when PRDX6 levels were restored. The study reveals that KLF9-PRDX6-ROS-NLRP3 forms an essential molecular network which connects OS to tissue damage and trophoblast dysfunction [35].

OS induced by H_2_O_2_ in HTR-8/SVneo trophoblasts activates both NLRP1 and NLRP3 inflammasomes, leading to increased caspase-1 activation and IL-1β maturation. This inflammatory response leads to elevated levels of LC3-II, Beclin-1, ATG5, and ATG7 proteins and decreased p62 protein levels. The suppression of NLRP1 activity leads to reduced inflammasome activation which results in decreased inflammatory cytokine production and increased autophagic activity that shows a compensatory link between inflammasome activation and autophagy during OS. Li M. et al. demonstrated that NLRP1 serves as a critical biological link which connects OS to inflammatory responses and autophagic regulation in human trophoblast cells [36].

In the study of Lu Y. et al. [37] it was proved that DCTPP1 functions as a vital regulator of redox homeostasis and survival in human extravillous trophoblasts. DCTPP1 gene silencing in HTR-8/SVneo cells led to increased ROS production which caused cells to develop higher lipid peroxidation and 4-HNE accumulation and resulted in severe OS. This redox imbalance was accompanied by impaired cell proliferation and a significant increase in apoptosis. Transcriptomic analysis revealed that DCTPP1 silencing alters the expression of genes involved in OS, apoptosis, and FOXO signaling pathways, including key regulators of ROS production and cell-cycle control. The study demonstrated that DCTPP1 binds to AUF1 which functions as a protective factor against OS. The AUF1 knockdown process generated results which were identical to DCTPP1 deficiency because it led to ROS accumulation and resulted in growth inhibition and apoptotic cell death. RNA-seq analysis showed that DCTPP1 and AUF1 proteins control identical gene expression patterns which control redox signaling and apoptosis, cooperating to protect trophoblast redox balance and viability.

TBH-induced OS in HTR-8/SVneo trophoblasts significantly increased lipid peroxidation and protein carbonylation without affecting cell viability. TBH suppressed trophoblast proliferation migration and culture growth while promoting apoptosis and reducing VEGF-A expression. The glucose uptake through GLUT1 showed no alterations. The antioxidant test revealed that α-tocopherol proved to be the only effective compound which restored both TBH-caused growth inhibition and oxidative damage in trophoblast cells. Pinto-Ribeiro L et al. linked lipid peroxidation and impaired trophoblast function [38].

The overexpression of CYP11A1 in BeWo trophoblasts results in excessive mitochondrial biogenesis via activation of the PGC1α-NRF1-TFAM axis which induces elevated levels of mitochondrial ROS and mtDNA damage. Mitochondrial dysfunction causes membrane potential loss and activation of mitochondrial apoptosis and results in elevated IL-6 production that creates a proinflammatory trophoblast phenotype. Conditioned medium from CYP11A1-overexpressing trophoblasts also suppresses both neural stem cell proliferation and causes DNA damage to these cells. The CYP11A1 enzyme produces mitochondrial OS which leads to harmful paracrine effects that spread beyond the placenta [39].

**Figure 1:**
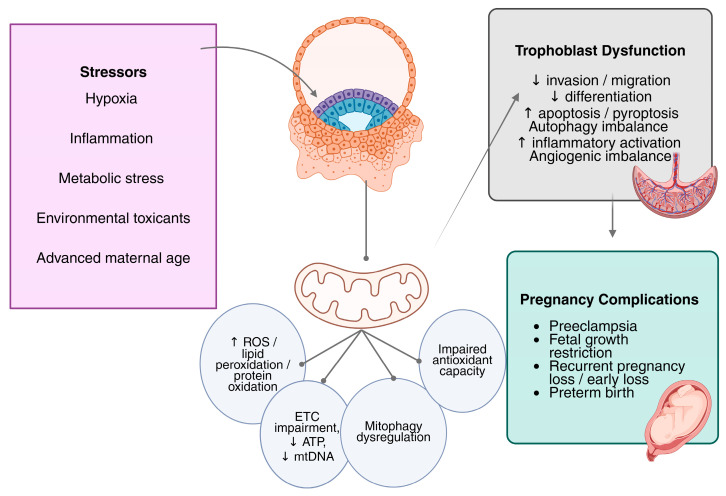
Mitochondria provide a unifying mechanistic framework linking diverse stressors to placental dysfunction. Trophoblast cell mitochondrial dysfunction is the result of several pregnancy-related stresses, including H/R, metabolic stress (hyperglycemia and dyslipidemia), inflammation, exposure to environmental toxicants, and advanced maternal age. Reduced ATP synthesis, changed mitochondrial dynamics, degraded mitochondrial DNA integrity, increased mitochondrial ROS generation, and impaired oxidative phosphorylation (ETC complex dysfunction) are the hallmarks of this dysfunction. Increased ROS triggers self-amplifying feedback loops that worsen OS and trigger inflammatory reactions, gene regulation mechanisms, and redox-sensitive signaling pathways. Ultimately, these interrelated mechanisms reduce trophoblast migration, proliferation, invasion, fusion, and angiogenic potential, thereby contributing to placental dysfunction in PE and associated pregnancy complications. Abbreviations: ROS, reactive oxygen species; ETC, electron transport chain; mtDNA, mitochondrial DNA; ATP, adenosine triphosphate; ↑, increased; ↓, decreased.

FABP5 is significantly downregulated in decidual stromal cells (DSCs) from recurrent spontaneous abortion, leading to mitochondrial dysfunction and intrinsic apoptosis through BAX, cytochrome c, and caspase-3 activation. Mechanistically, FABP5 maintains mitochondrial structure through its ability to control MRPL17 which functions as a mitochondrial ribosomal protein needed for OXPHOS protein translation and ATP synthesis. The FABP5-MRPL17 signaling pathway controls DSCs to release CXCL11 while its disruption blocks CXCR3-MMP-2/MMP-9 signaling in trophoblasts which results in impairing trophoblast migration and invasion [40]. Taken together, these diverse maternal, metabolic, inflammatory, and environmental stressors converge on mitochondrial dysfunction and OS as common mechanisms underlying placental maladaptation. A summary of the major placental stressors, associated mitochondrial alterations, and trophoblast functional outcomes is provided in Table 1. Figure 1 summarizes the mechanistic framework linking diverse stressors to OS and ultimately to placental dysfunction.

## 4. Discussion

Evidence shows that trophoblast mitochondrial dysfunction is not always proportional to mitochondrial protein abundance. During hypoxia, mitochondrial content may decrease but often recovers after reoxygenation, whereas mitochondrial respiratory function remains persistently impaired. This dissociation indicates that hypoxia causes permanent mitochondrial dysfunction rather than transient organelle loss, rendering trophoblast cells more vulnerable to oxidative damage and energetic failure [41].

Mitochondrial dynamics strongly influence trophoblast responses to OS. The experimental induction of OS induces DRP1 Ser616 phosphorylation, promoting excessive mitochondrial fission, loss of membrane potential, and activation of apoptotic pathways. These findings suggest that dysregulated mitochondrial dynamics can shift quality-control processes toward cell death, particularly under excessive or prolonged stress, underscoring the context-dependent effects of fission–fusion regulation [42].

In all of the examined investigations, trophoblast mitochondrial dysfunction is predominantly qualitative rather than purely quantitative. Reduced ATP production, loss of mitochondrial membrane potential, damaged mitochondrial DNA integrity, and impaired oxidative phosphorylation complex activity (CI–CV) are more consistent results, while declines in mitochondrial content are occasionally noted [43]. A metabolic shift toward glycolysis often accompanies these bioenergetic abnormalities, indicating an adaptive response that becomes inadequate under prolonged OS. Crucially, mitochondrial bioenergetic failure is directly linked to placental dysfunction. These mitochondrial impairments are associated with altered trophoblast behaviors, including decreased invasion, impaired fusion, reduced differentiation capacity, and increased apoptotic susceptibility [44].

Trophoblast antioxidant adaptation also appears limited under pathological OS. Although Nrf2 and its downstream effector HO-1 are frequently upregulated in hypoxia–reoxygenation models and in preeclamptic placental tissues, this activation often occurs after antioxidant enzyme activity has already declined. This paradox suggests that Nrf2 pathway activation represents a compensatory response that is insufficient to restore redox equilibrium. Accordingly, Nrf2 activation in diseased placentas likely reflects established oxidative injury rather than complete protection, helping to reconcile contradictory reports regarding its role [45].

Mitophagy emerges as another critical mechanism which maintains trophoblast structure but remains sensitive to OS levels. BNIP3 expression levels are decreased in preeclamptic placentas, resulting in impaired mitophagic removal of damaged mitochondria. This leads to the accumulation of dysfunctional organelles, increased ROS production, and enhanced trophoblast cell death. Defective BNIP3-dependent mitophagy therefore compromises mitochondrial quality control and contributes to impaired trophoblast migration and invasion, ultimately leading to placental functional defects. Notably, both excessive and insufficient mitophagy can exacerbate OS, indicating that its net effect depends on the magnitude, duration, and cellular context of the insult [46].

Hypoxia-related signaling in pathological placentas can also diverge from physiological placental development. HIF-1α and HIF-2α are increased in PE and IUGR, despite apparently intact oxygen-sensing machinery. In this context, impaired degradation of HIF-α—due to competition between DJ-1 and the VHL complex—may lead to persistent HIF activation. This posttranslational dysregulation suggests that hypoxic signaling in PE arises from altered regulatory control rather than appropriate environmental sensing. Such divergence may explain why HIF signaling can drive maladaptive metabolic and angiogenic responses in disease while supporting normal placentation early in gestation [47].

OS can also induce epigenetic modifications that include both transient and durable changes. Hypoxia-driven histone hypermethylation leads to a sustained reduction in antioxidant enzyme expression, thereby linking redox stress to chromatin modification. Such epigenetic reprogramming provides a mechanistic explanation for why OS-induced damage persists even after removal of the initial insult, leading to ongoing placental dysfunction [48].

Emerging transcriptomic analyses of syncytiotrophoblast-derived extracellular vesicles implicate ferroptosis-related pathways in PE. Gene expression analyses indicate that OS, hypoxia, iron regulation, and NADPH oxidase activity contribute to redox imbalance through distinct mechanisms. These pathways do not follow classical apoptotic signaling. However, because these observations are largely in silico, experimental validation is required. At present, ferroptosis-related signatures should be interpreted as emerging and hypothesis-generating rather than established drivers of placental dysfunction [49].

Mitochondrial OS is a shared feature across multiple placental stress contexts, including gestational diabetes, fetal growth restriction, environmental toxicant exposure, advanced maternal age, and hypoxia-driven placental insufficiency. However, this review focuses primarily on PE due to the breadth and consistency of available evidence [50]. Although the stressors that cause these situations vary, they have mitochondrial markers, including reduced bioenergetics, elevated ROS generation, and altered redox signaling. Disease-specific variations are also apparent; hypoxia and PE more substantially affect angiogenic balance and mitochondrial dynamics, while metabolic stress in gestational diabetes primarily modifies glucose management and insulin signaling [51,52]. Importantly, findings derived from transformed trophoblast cell lines, primary trophoblasts, placental explants, and patient tissues should not be interpreted as interchangeable, as each model captures distinct aspects of trophoblast biology and stress responsiveness [53].

Finally, the functional consequences of OS are reflected in distinct patterns of trophoblast behavioral impairment. Ischemia–reperfusion injury demonstrates that OS suppresses trophoblast migration and proliferation through ROS-mediated pathways, but it impairs invasion by ROS-independent downregulation of MMP-9. These results highlight the intricacy of placental stress responses by showing that OS regulates trophoblast activities differently depending on the molecular mechanism involved [54].

As illustrated in Figure 1, diverse placental stressors converge on mitochondrial dysfunction, leading to bioenergetic failure, increased ROS, and self-sustaining feedback loops involving inflammation, redox signaling, and gene regulation that ultimately impair trophoblast function. Collectively, these findings indicate that OS-responsive mechanisms, including mitophagy, Nrf2 signaling, HIF activation, autophagy, mitochondrial dynamics, and epigenetic regulation, are not uniformly beneficial or harmful. Instead, their effects depend on trophoblast subtype, gestational age, stress magnitude and duration, and whether exposure is acute or chronic. This context-dependent framework helps explain variability across experimental models and reconciles seemingly contradictory findings in the literature.

## 5. Conclusions

Overall, the evidence in this review shows OS functions as a primary factor which causes placental dysfunction that leads to PE and additional pregnancy complications. Importantly, OS emerges as a multifaceted process which shows direct connections to mitochondrial damage. Across different experimental models and clinical samples, it is demonstrated that mitochondrial bioenergetics and dynamics become affected through three main mechanisms: impaired antioxidant defenses, inflammatory activation, and epigenetic alterations.

The combination of hypoxia, inflammation, and metabolic and environmental stress leads to mitochondrial dysfunction which results in undermining trophoblast viability, migration, invasion, and differentiation. The evidence suggests that stress-response pathways including Nrf2 and HIF signaling become activated but their compensatory responses fail to achieve placental homeostasis restoration. The system continues to experience oxidative damage because mitochondrial dysfunction and abnormal redox signaling create a self-perpetuating cycle which damages trophoblast cells.

These observations indicate that OS plays a central role in disease progression, given the pivotal role of mitochondria in regulating cellular function. Research should focus on mitochondrial function and redox balance to enhance placental health and pregnancy results according to this perspective of placental OS.

## Figures and Tables

**Table 1:** Summary of mitochondrial stress mechanisms and trophoblast outcomes in placental pathologies. This table provides a conceptual overview of recurrent mitochondrial stress mechanisms and trophoblast functional effects discussed in this review.

Stress Context	Mitochondrial Alteration	Key Trophoblast Outcome	Key Limitation/Gap
PE	Impaired OXPHOS, increased ROS	Reduced invasion, apoptosis	Causality unresolved
Hypoxia/H-R	Mitochondrial fission, OS	Altered differentiation	Model-dependent effects
GDM	Metabolic reprogramming	Insulin resistance	Human validation limited
Toxicants	mtDNA damage	Senescence/apoptosis	Exposure heterogeneity

## Data Availability

No new data were created or analyzed in this study.

## Abbreviations

The following abbreviations are used in this manuscript:

ABCA1	ATP-binding cassette subfamily A member 1
ABCG1	ATP-binding cassette subfamily G member 1
AUF1	AU-rich element RNA-binding protein 1
ATP	Adenosine triphosphate
AMA	Advanced maternal age
AOPPs	Advanced oxidation protein products
AGTR4	Angiotensin II receptor type 4
ANKRD37	Ankyrin repeat domain containing 37
ATG5	Autophagy related 5
ATG7	Autophagy related 7
Bcl-2	B-cell lymphoma 2
Bax	BCL2 associated X, apoptosis regulator
BNIP3	BCL2 interacting protein 3
BNIP3L	BCL2 interacting protein 3-like
BECN1	Beclin1
CXCR3	C-X-C motif chemokine receptor 3
CXCL11	Chemokine (C-X-C motif) ligand 11
CGB	Chorionic gonadotropin subunit beta
COLEC12	Collectin subfamily member 12
cAMP	Cyclic adenosine monophosphate
p21	Cyclin-dependent kinase inhibitor 1A
CYP11A1	Cytochrome P450 family 11 subfamily A member 1
CYP19A1	Cytochrome P450 family 19 subfamily A member 1
COX	Cytochrome c oxidase
COXII	Cytochrome c oxidase subunit II
CTB	Cytotrophoblast
DNMT1	DNA methyltransferase 1
DNMT3A	DNA methyltransferase 3 alpha
DSCs	Decidual stromal cells
DCTPP1	Deoxycytidine triphosphate pyrophosphatase 1
DNM1L	Dynamin 1-like
DRP1	Dynamin-related protein 1
ETC	Electron transport chain
ER	Endoplasmic reticulum
EGFR	Epidermal growth factor receptor
ERRα	Estrogen-related receptor alpha
EVs	Extracellular vesicles
EVT	Extravillous trophoblast
FABP5	Fatty acid binding protein 5
FLT-1	Fms related receptor tyrosine kinase 1
FOXO	Forkhead box O
GDM	Gestational diabetes mellitus
GLUT1	Glucose transporter 1
GLUT4	Glucose transporter 4
GRP78	Glucose-regulated protein 78
GRP94	Glucose-regulated protein 94
GCLC	Glutamate-cysteine ligase catalytic subunit
GSH	Glutathione
GSH-Px	Glutathione peroxidase
HSPB8	Heat shock protein family B (small) member 8
HO-1	Heme oxygenase 1
HMOX-1	Heme oxygenase 1 (gene)
HKII	Hexokinase II
hAECs	Human amniotic epithelial cells
hCG	Human chorionic gonadotropin
HIP	Hyperglycemia in pregnancy
HIF-1α	Hypoxia-inducible factor 1 alpha
HIF-2α	Hypoxia-inducible factor 2 alpha
iPSCs	Induced pluripotent stem cells
IRE1α	Inositol-requiring enzyme 1 alpha
IRβ	Insulin receptor beta
IRS-1	Insulin receptor substrate 1
IL-1β	Interleukin 1 beta
I/R	Ischemia–reperfusion
Keap1	Kelch-like ECH-associated protein 1
KLF9	Krüppel-like factor 9
LAT1	L-type amino acid transporter 1
LDLR	Low-density lipoprotein receptor
MDA	Malondialdehyde
MMP-2	Matrix metalloproteinase 2
MMP-9	Matrix metalloproteinase 9
MGST1	Microsomal glutathione S-transferase 1
mtDNA	Mitochondrial DNA
FtMt	Mitochondrial ferritin
mtROS	Mitochondrial reactive oxygen species
MEHP	Mono-(2-ethylhexyl) phthalate
MPO	Myeloperoxidase
NAC	N-acetylcysteine
NOX4	NADPH oxidase 4
NLRP1	NLR family pyrin domain containing 1
NLRP3	NLR family pyrin domain containing 3
NRF2	Nuclear factor erythroid 2-related factor 2
NR4A2	Nuclear receptor subfamily 4 group A member 2
OS	Oxidative stress
PRDX1	Peroxiredoxin 1
PRDX6	Peroxiredoxin 6
PTEN	Phosphatase and tensin homolog
PGM5	Phosphoglucomutase 5
PlGF	Placental growth factor
PE	Preeclampsia
PGE2	Prostaglandin E2
COX-2	Prostaglandin-endoperoxide synthase 2
AKT	Protein kinase B
ROS	Reactive oxygen species
RND3	Rho family GTPase 3
p70S6K	Ribosomal protein S6 kinase beta-1
SR-BI	Scavenger receptor class B type I
SA-β-gal	Senescence-associated beta-galactosidase
SAHF	Senescence-associated heterochromatin foci
SPINT1	Serine peptidase inhibitor, Kunitz type 1
SIRT3	Sirtuin 3
SLC1A5	Solute carrier family 1 member 5
SOD	Superoxide dismutase
TRX	Thioredoxin
TIMP-1	Tissue inhibitor of metalloproteinases 1
TIMP-2	Tissue inhibitor of metalloproteinases 2
TNF	Tumor necrosis factor
UCP2	Uncoupling protein 2
uPA	Urokinase-type plasminogen activator
VEGF-A	Vascular endothelial growth factor A
VHL	Von Hippel–Lindau tumor suppressor
YAP	Yes-associated protein
p38MAPK	p38 mitogen-activated protein kinase
MnSOD2	Manganese superoxide dismutase 2
